# Editorial: Interaction Between Hyaluronic Acid and Its Receptors (CD44, RHAMM) Regulates the Activity of Inflammation and Cancer

**DOI:** 10.3389/fimmu.2016.00039

**Published:** 2016-02-08

**Authors:** David Naor

**Affiliations:** ^1^Lautenberg Center of Immunology, IMRIC, Faculty of Medicine, Hebrew University of Jerusalem, Jerusalem, Israel

**Keywords:** CD44, RHAMM, hyaluronan, inflammation, cancer

**The Editorial on the Research Topic**

**Interaction Between Hyaluronic Acid and Its Receptors (CD44, RHAMM) Regulates the Activity of Inflammation and Cancer**

An old Indian legend tells a story about six blind men who touched an elephant. The first man who touched his leg said: “it is a pillar.” The second man who touched the tail said: “it is a rope.” The third man who touched the trunk of the elephant said: “it is a thick branch of a tree.” The fourth man who touched the ear said: “It is a big hand fan.” The fifth man who touched the belly said: “It is a huge wall.” The sixth man who touched the tusk of the elephant said: “It is a solid pipe.” Each one of them loudly insisted that his claim is the right one. Then wise old man arrived to the place of scene, listened to their arguments and said “all of you are right, but all together you identified an elephant.”

Similarly, each one of the 18 chapters of this e-book tells a different, fascinating story about the “biological polygamy” of hyaluronan and its receptors. Yet, this story is focused on the author’s specific field of interest or discipline. On the other hand, when the 18 chapters are collected in one e-book, each of them fosters the others and collectively a complete scene is created.

Hyaluronan or hyaluronic acid (HA), which resides in the interstitial collagenous matrices, increases viscosity and hydration and binds to a “link module motif” of HA-binding proteoglycans (e.g., CD44) and link proteins. HA is a non-sulfated, linear glycosaminoglycan (GAG) composed of repeating disaccharides of (β, 1–4)-glucuronic acid (GlcUA) and (β, 1-3)-N-acetyl glucosamine (GlcNAc). In most tissues, native HA has a high molecular mass of 1000–10,000 kDa, with extended molecular lengths of 2–20 μm. HA plays crucial roles in structuring tissue architecture, in cell motility, in cell adhesion, and in proliferation processes (1, 2).

Hyaluronic acid is synthesized by three HA synthase (HAS) proteins. These generate predominantly high molecular weight-HA (HMW-HA) of between 200 and 2000 kDa. HA catabolism is mediated by hyaluronidases, mechanical forces, and oxidative stress (reactive oxygen and nitrogen species). The degradation generates different-sized HA polymers (or fragments), abbreviated low molecular weight-HA (LMW-HA; <200 kDa) and HA oligomers (1).

In general (exceptions do exist), LMW-HA is pro-inflammatory and pro-cancerous, whereas HMW-HA is anti-inflammatory and anti-cancerous. In this context, a vicious cycle is generated. Inflammatory conditions activate the production of HAS, which synthesizes HA. Subsequently, the HA is degraded by hyaluronidases and reactive oxygen species, the generation of which is also induced by the inflammation. The resulting cleaved HA fragments further propagate the inflammation. This perpetuating cycle can be blocked by competition with excess HMW-HA. To this end, Tian et al. found that naked mole-rat fibroblasts secrete HMW-HA, which is over five times larger (6000–12000 kDa), than human or mouse HA (500–2000 kDa). The HMW-HA accumulates in naked mole-rat tissues. This rodent has a lifespan exceeding 30 years and is resistant to cancer. Interestingly, once HMW-HA is removed by knocking down HAS-2, or by overexpressing hyaluronidase 2, which cleaves HMW-HA, the naked mole-rat cells become susceptible to malignant transformation and form tumors (3). Notably, the pro-inflammatory role of LMW-HA, including HA fragments and oligo-HA, displays not only pathological effects but eventually also physiological activities, such as expression of β-defensins to combat microbial infections (4) or induction of inflammation to accelerate wound healing (5).

CD44 glycoprotein is expressed on the surface of many mammalian cells, including leukocytes, endothelial cells, epithelial cells, fibroblasts, and keratinocytes. Extensive alternative splicing of nine variable exons and distinct post-translational modifications generate many CD44 isoforms. Standard CD44 is the smallest and most abundant isoform, whereas the other variants are expressed in a cell-specific manner (e.g., on epithelial cells or keratinocytes), as well as in multiple pathologies, including rheumatoid arthritis, diabetes, multiple sclerosis, and cancer (2, 6). The physiological activities of CD44 stem from its multiple functions, including mediating cell–cell and cell–matrix interactions, cell proliferation, cell adhesion, cell migration, hematopoiesis, lymphocyte activation, cell homing, cell extravasation, cell survival, and apoptosis, as well as epithelial–mesenchymal transition (EMT) (7). However, these functions can be converted to pathological activities when exaggerated or they escape out of control, like in cancer or chronic inflammation. Most of the CD44 studies are limited to preclinical models. However, the use of anti-CD44 antibodies in a few clinical trials resulted in life-threatening toxicity (8). Therefore, the risks vs. the benefits must be carefully evaluated before CD44-targeting strategies are translated to the clinic.

The receptor for HA-mediated motility (RHAMM or CD168), such as CD44, is also alternatively spliced, albeit at a much lower intensity. Variant forms of RHAMM are found on both cell surfaces and inside the cells (9). However, unlike CD44, RHAMM isoforms do not have the link module domain. Instead, they have a BX7B motif that binds HA, where “B” represents arginine or lysine, and “X” represents any non-acidic amino acid (10). RHAMM supports both malignancy and wound healing processes.

As CD44 supports both chronic inflammation and cancer progression in many (but not all) experimental models and human diseases, CD44 targeting, e.g., by antibody, was successfully documented in many preclinical studies, such as collagen-induced arthritis (CIA) (11). Surprisingly, we found that CD44 targeting by CD44 gene deletion in the embryo aggravates CIA, rather than ameliorating it. It appears that a CD44 redundancy process in the CD44 deleted embryo allows up-regulation of RHAMM, which replaces CD44 also during adulthood. The substituting RHAMM supports CIA joint inflammation more effectively than CD44 (11), because it is a better supporter of cell migration. It is not surprising that CD44 targeting in the adult is not redundant, like in the embryo, as CD44 in the embryo displays a survival-supporting function that generates pressure for ultimate RHAMM replacement. Such a developmental pressure does not exist in the adult, so that CD44 targeting is not compensated by functional RHAMM at this phase of life, and the therapeutic effect by CD44 targeting, can be documented.

TLR-4, a principle innate receptor of bacterial LPS, is also an important receptor of HA (1). TLR-4 activates nuclear factor (NF)-κB protein via two major routes: a myeloid differentiation factor (MyD) 88-dependent pathway that acts via NF-κB to induce pro-inflammatory cytokines and a MyD88-independent pathway that acts via type I interferons to increase the expression of interferon-inducible pro-inflammatory genes.

Siiskonen et al. describe the mysterious and unexpected functions of hyaluronan synthase 1 (HAS-1), which is less known and less explored than its two HAS-2 and HAS-3 enzyme “step brothers,” which also engage in HA synthesis. As HAS-1 embryonic gene deletion does not influence the normal phenotype, this raises the questions: are its functions compensated (by redundancy) by the two other HAS enzymes, and if so, why has HAS-1 has been preserved in the course of evolution?

Receptor (e.g., CD44) sensitivity to hyaluronan quantity and size provides a biosensor of the state of the microenvironment (inflammation, cancer stroma, or wound healing) surrounding the cell. Hence, to learn more on the chemical profile of HA in the context of these parameters or on technologies associated with its quantification, specification, isolation, and size determination in both fluids and tissues, it would be highly beneficial to read Cowman et al. communication.

Readers interested in the structural alternations associated with the HA-binding domain (HABD) of CD44 after HA binding, cannot miss Guvench’s chapter. The authors (Guvench et al.) performed extensive all-atom explicit-solvent molecular dynamics (MD) simulations of HABD and the conclusions are presented in this communication. However, the HABD was analyzed independently of the rest of the CD44 molecule, while the transmembrane domain and especially the cytoplasmic tail influence the binding affinity as well (2, 6). Furthermore, it should be recalled that in this study, the conclusions are limited to HABD interaction with HA oligomers, whereas larger HA molecules were not evaluated.

Monslow et al. comprehensively reviewed the role of HA in health and disease, especially in relation to HA size. Their size definition for HA is formulated as follows: HMW-HA: >1000 kDa; intermediate (medium) molecular weight-HA (MMW-HA): 250–1000 kDa; LMW-HA: >10–250 kDa; and oligo-HA (<10 kDa). However, there is no consensus on these definitions and standardization of these values by an international workshop is necessary. In general, there is a consensus that HMW-HA controls normal homeostasis and displays anti-inflammatory and anti-cancerous effects, with a few exceptions. Many researchers consider LMW-HA and oligo-HA pro-inflammatory and pro-cancerous GAGs, as well as stimulators of pro-inflammatory cytokines. Yet, there are many contradictory findings. This confusion is related to the lack of consensus on size definition, polydispersity of HA commercial products (different HA sizes in the same product), the use of HA from different animal sources or from different tissues, and, finally, the impurity of commercial products. These reservations must be taken into account whenever a new study on HA is undertaken.

Four-methylumbelliferone (4-MU) is an HA-antagonizing product, described by Nagy et al. The product inhibits HAS synthesis by reducing the availability of UDP-GlcUA to the enzyme, thus, interfering with HA synthesis and consequently with HA-related pathologies, such as cancer and autoimmunity. As 4-MU is an already approved drug called “hymecromone” for biliary spasm, the road to 4-MU therapy of inflammatory diseases and malignancy has been largely paved.

Hyaluronan and CD44 reside in the lipid rafts, cholesterol- and glycosphingolipid-enriched membrane microdomains that regulate the membrane receptors as well as signal delivery from the cell surface into the cell. Murai et al. examines in particular lipid raft regulation of HA binding to the CD44 of T lymphocytes and malignant cells, binding, which leads to rolling interactions on vascular endothelial cells, an important phase in inflammation and cancer development.

If the reader centers his/her interest on the inter-relationship between the tumor and its inflammatory microenvironment in context to HA, he/she can be referred to the article by Nikitovic et al. The authors focus their discussion on the influence of the cancer inflammatory environment on tumor growth, with specific emphasis on stromal HA.

The interplay between the tumor and its stromal microenvironment is also documented by Schwertfeger et al., using breast cancer as an example. The generation of a pro-tumorigenic inflammatory environment during breast cancer development requires LMW-HA-induced recruitment and activation of inflammatory macrophages. The macrophages release NFkB-regulated pro-inflammatory factors (IL-1β, IL-12, reactive oxygen species), normally involved in tissue repair. Hence, the cancer cells “stole” the inflammation supportive machinery from the wound healing process.

Such inter-relationships between the tumor and its microenvironment are described also in hematological tumors. Gutjahr et al. call our attention to the pro-cancerous survival (or anti-apoptotic) signals delivered by the tumor inflammatory environment, focusing on acute lymphocytic leukemia (CLL). Long-term survival and proliferation of CLL cells requires their dynamic interaction with stromal and immune cells in lymphoid organs. Interactions of HA with cell surface CD44 or RHAMM contribute to CLL cell localization, and hence to CLL pathophysiology. Deep mining of these complex interactions may reveal links more susceptible to therapeutic targeting, such as CD44v6, RHAMM, VLA-4, ZAP-70, or HAS (for details, see this communication).

Lee-Sayer et al. focus their attention on the inter-relationships between HA and CD44 in cells involved in the innate and adoptive immune system in the context of inflammation. Under innate inflammatory conditions, dendritic cells express HA on their membrane and T cells upregulate CD44. In the adoptive phase, interactions between the HA of the antigen-presenting dendritic cells and the activated CD44 of T lymphocytes may allow intimate contact between the co-stimulating molecules of the former and accessory molecules of the latter, leading to activation of the lymphocyte’s T cell receptor. Going one step further, the HA and the CD44 molecules may also be considered co-stimulating and accessory molecules.

If the reader wants to know how HMW-HA and LMW-HA are involved in allergic inflammation, he/she should focus on the communication by Kim et al. The reader can surmise, following extrapolation from the inflammation data, that HMW-HA is anti-allergic, whereas LMW-HA is pro-allergic. The mechanisms underlying these effects, including the role of microRNAs, are reported in detail.

McDonald and Kubes describe the cell trafficking roles on endothelial cells in the liver, which are different than those in other tissues. Recent evidence implicates serum-derived hyaluronan-associated protein (SHAP) as an important co-factor that strengthens the binding of HA to CD44 under shear stress, resulting in improved cell extravasation. Finally, the authors indicate that HA–CD44 interaction supports not only destructive chronic inflammation but also the trafficking of stem cells that resolve the inflammation, the balance between the two determining the tissue’s fate.

Bourguignon and Bikle suggest that the interaction of large HA (>1000 kDa) with cell surface CD44 leads to Rac-signaling and normal keratinocyte differentiation, DNA repair, and survival function. On the other hand, the interaction of small/fragmented (10–100 kDa) HA (generated by UV irradiation) with cell surface CD44 stimulates RhoA/ROC activation, NFκB/Stat-3 signaling, and microRNA-21 production, resulting in proliferation and inflammation, as well as in the progression of squamous cell carcinomas (SCC). A balance that favors the “good” Rac-signaling over the “bad” RhoA signaling can be generated by Y27623, a ROK inhibitor, vitamin D, or by triggering HAS-2, which activates the production of large HA. These therapeutic approaches may be used for therapy of patients with UV irradiation-skin diseases (for more details, see the article).

Misra et al. comprehensively describe technologies that can be used to modulate the signals of HA–HA receptor interactions in favor of the patient. A sophisticated approach is Misra’s technology relating to transferrin-coated nanoparticles, which include CD44v6 shRNA, to silence the CD44v6 gene in tumor cells expressing transferrin receptor. Readers, who seek information on this fascinating approach, or to other therapeutic strategies based on disrupting HA–CD44 interactions and subsequent signaling, are invited to read this chapter.

The use of a CD44-targeting peptide, Ac-KPSSPPEE-NH2, is another therapeutic strategy, documented by Finlayson, to combat CD44-associated pathological activities in experimental vascularized eye, tumor xenografts, or in clinical trials. If the reader wishes to know more on the peptide’s mechanism of action, it is recommended to read this chapter.

Jordan et al. focuses our attention on normal and aberrant cellular signaling generated after interaction of HA with its receptor (mainly CD44), under different physiological and pathological settings. These include bacterial infection, viral infection, interstitial lung disease, wound healing, chronic inflammation (autoimmunity), and cancer. The outcome of such aberrant signaling is uncontrolled cell migration, cell proliferation, cell survival (e.g., of cancer cells), apoptosis [e.g., of β cells in type 1 diabetes; (12)], angiogenesis, and EMT, leading to different pathologies.

Both hematopoietic stem cells (HSCs) and leukemia stem cells (LSCs), also known as leukemia-initiating cells (LICs) seek a “shelter” called a bone marrow “niche.” The niche maintains the “ stemness” of the host cells, i.e., supports their survival and homing as well as regulates the balance between their quiescence and growth. Once HSCs are transplanted into a leukemic patients, they eventually compete with LICs for lodging in the niche, engaging their cell surface CD44 in interaction with the HA of the niche. In this communication, Zöller raises the question: how can an advantage be imparted to the transplanted HSCs over the patient’s LICs in the context of HA–CD44 interaction, in view of their largely identical biological nature, when they compete for “shelter” in the same niche. The answer to the question may be found in this communication.

Orian-Rousseau’s communication is focused on the role of CD44 isoforms as co-receptors, especially for receptor tyrosine kinases (RTK). She further calls our attention to the involvement of CD44 in Wnt signaling, both as a regulator of the Wnt receptor (via interaction with LRP6) or as a Wnt target gene, e.g., for CD44v6 or Met-RTK expression. Involvement of CD44 in Wnt signaling, leading to EMT, is also discussed. Finally, Dr Orian-Rousseau speculates on the function of CD44 in cancer stem cells (CSCs), which has so far has been studied as a biomarker for these cells, but its role in CSCs remains elusive. Integration of the CD44v6 co-receptor (activated by HA ?) and Met-RTK (activated by hepatocyte growth factor) with Wnt signaling may explain what could be the role of CSC CD44 in colorectal cancer and perhaps other malignancies, i.e., by promotion of cell migration and metastasis.

In conclusion, the elephant unveiled in this e-book reveals a fascinating story about the HA–CD44 interaction, which not only exposes the underlying mechanism of this interaction but also allows identification of weak links, which can be targeted by various therapeutic approaches in both cancer and inflammatory diseases.

## Author Contributions

The author confirms being the sole contributor of this work and approved it for publication.

## Conflict of Interest Statement

The author declares that the research was conducted in the absence of any commercial or financial relationships that could be construed as a potential conflict of interest.
